# Eye centring in selfies posted on *Instagram*

**DOI:** 10.1371/journal.pone.0218663

**Published:** 2019-07-17

**Authors:** Nicola Bruno, Marco Bertamini, Christopher W. Tyler

**Affiliations:** 1 DiMeC, Università di Parma, Parma, Italy; 2 Department of Psychological Science, University of Liverpool, Liverpool, United Kingdom; 3 Division of Optometry and Vision Sciences, School of Health Sciences, City University of London, London, United Kingdom; University of Sydney, AUSTRALIA

## Abstract

Earlier work by one of us examined a historical corpus of portraits and found that artists often paint the subject such that one eye is centred horizontally. If due to psychological mechanisms constraining artistic composition, this eye-centring bias should be detectable also in portraits by non-professionals. However, this finding has been questioned both on theoretical and empirical grounds. Here we tested eye-centring in a larger (N ~ = 4000) and more representative set of selfies spontaneously posted on *Instagram* from six world cities. In contrast with previous selfie results, the distribution of the most-centred eye position peaked almost exactly at the horizontal centre of the image and was statistically different from predictions based on realistic Monte-Carlo predictions. In addition, we observed a small but statistically reliable pseudoneglect effect as well as a preference for centring the left-eye. An eye-centring tendency appears to exist in self-portraits by non-artists.

## Introduction

How do visual artists compose their work? Rudolf Arnheim [1] famously argued that a key mechanism in pictorial composition is the dynamics of *centric* and *eccentric* perceptual “forces”. Thus, he proposed that artistic composition is shaped by tradeoffs between forces applied in relation to variously defined centres within pictorial structures. Perhaps one of the best candidate examples of compositional dynamics in relation to centres is an observation reported by Tyler [2–3]. Examining a corpus of portraits and self-portraits from various sources, Tyler observed that most had one eye positioned close to the vertical centre line. Eye-centring may be considered a trade-off between a centric force as applied to one key element of the composition (the eyes) and other forces in play amongst the various compositional elements, such as the face as a whole. Tyler [2] suggested that horizontal eye-centering at a location above the vertical center represents a hidden principle of composition which painters have applied either consciously or unconsciously throughout the centuries.

The validity of an eye-centring principle in visual composition has been questioned by two lines of argument. The first is theoretical. Based on a Monte Carlo simulation, McManus & Thomas [4] have suggested that eye centring may be a statistical artifact. Given a random process—they argued—the distribution of the most-centred eye may well peak at the horizontal centre due to constraints that apply when a relatively large object, such as a head, must be fitted within the picture frame. In addition, they argued that if a preference for centring one eye was at play in composition, then the peak of the distribution should not be at the horizontal centre of the image, but slightly displaced to the left of it due to pseudoneglect, the tendency of most non-pathological observes to mislocalize the perceived centre of an extent slightly to the left of its true centre [5]. Although important, neither of these objections is conclusive. The outcome of a Monte Carlo simulation is, by definition, determined by the parameters of the simulation itself and especially by the theoretical probability distribution used to model random error. If these are not realistic, results of the simulation are difficult to interpret [6]. With regard to pseudoneglect, it should be noted that the leftward error that defines this phenomenon is typically observed in specific tasks, such as line bisection, and is generally rather small (by most estimates, averaging around 1% of the bisected extent), very variable between individuals and tasks [7], and modulated by a host of mediating factors [8]. Whether one would predict it to be observed for eye placement within a portrait is therefore not obvious. The second argument against eye-centring has been empirical. Bruno, Gabriele, Tasso & Bertamini [9] examined a database of “selfies”, photographic self-portraits taken with a smartphone by untrained individuals, and found no evidence for eye-centring. The selfies in this study, however, were not photos freely taken for the purpose of being shared on social media (as is typical of selfies proper) but images produced on demand by laboratory participants who adhered to precise instructions on how to hold the phone and select a pose. It is possible therefore that this dataset failed to tap into the perceptual processes that govern visual composition in a realistic selfie-taking context.

Here we tested eye centring in a large and more representative set of selfies posted spontaneously on *Instagram*. Given the popularity of selfies as a phenomenon, large databases have become available. We used the *selfiecity* database, which includes selfies from six cities from different countries spanning the globe (for examples of these publicly available images, see www.selfiecity.net). In contrast with previous results, we did find evidence for eye centring in this dataset. The distribution of the most-centred-eye positions peaked almost exactly at the horizontal centre of the image. In addition, the distribution of most-centred eye positions proved narrower than a normal distribution (arguing against the simplest random-process model) and different in shape from that predicted by a Monte Carlo simulation (arguing against a more realistic random-process model). In addition, the mean horizontal position of the most-centred eye turned out to be slightly but significantly displaced to the left, as one would expect from pseudoneglect. We discuss these findings in the context of differences between artists and non-artists, and between on-demand laboratory selfies and selfies spontaneously posted on social media.

## Methods

### Hypothesis

We performed an observational study using a publicly available database of selfies, photographic self-portraits spontaneouly posted by untrained individuals on a social media site (see following section). If the eye-centring bias observed by Tyler reflects a general tendency in the composition of self-portraits, one would expect to observe it also in images created by individuals without academic training in painting or photography.

### Database

We used the publicly available *selfiecity* database (www.selfiecity.net), which consists of 3840 images spontaneously posted on the social media platform *Instagram* from six world cities (Bangkok, Berlin, London, Moscow, New York City, and São Paulo) from September 21 to 27, 2015 (London) and from December 4 to 12, 2013 (the other five cities). Use of the images complied with the terms and conditions of both websites. The original database was selected for the purposes of a social data visualization project by a group led by Lev Manovich at the City University of New York [10]. The selection used a combination of automatic face recognition and ratings provided by human observers, yielding a set of 640 images per city.

### Data validation

Due to imperfections in the selection process, the *selfiecity* database contains some images that are not selfies, as already noted in previous papers using this database [11–12]. Thus, all images that could not be considered selfies were excluded from further analysis. These included smartphone self-portraits portraying more than one individual (sometimes called *wefies*, *groupies*, or *usies)*, self-portraits of the sitter next to pets or life-sized dolls, and occasional images showing that the sitter had in fact been photographed by someone else (for instance, where the sitter’s pose revealed that he or she was not holding a camera). Occasional self-portraits in extreme poses, such as full profile poses, or head tilts exceeding 45 degrees, were also excluded. This resulted in the exclusion of 284 images, resulting in a validated database totaling 3556 usable selfies. Self-portraits of sitters wearing eyeglasses (especially sunglasses) were kept as well as self-portraits of sitters with eyes closed. The former images were inspected carefully to determine the position of the eye, whereas in the latter the centre of the eye was recorded by determining the mid-point along the line of the eyelashes.

### Dependent variable

**Fig 1:**
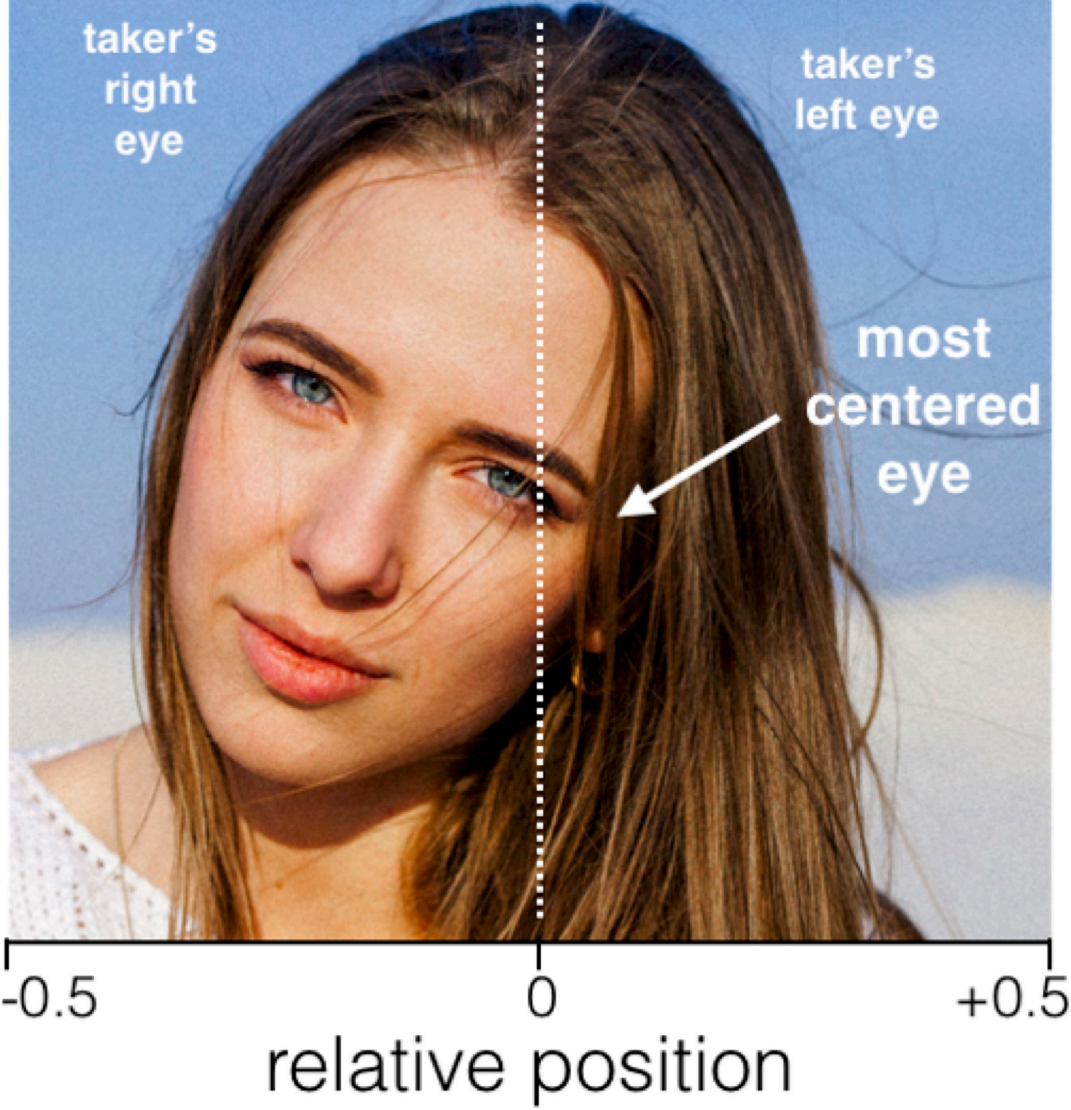
Criteria for measuring the relative position of the most-centred eye. The image was not in the examined database and was cropped for the purpose of illustrating measuring criteria. Image in the public domain (see www.pexels.com/photo-license).

We examined the distribution of the position of the most-centred eye in the selfies. In each selfie, this position was estimated by determining the position of the horizontal centre of the picture and those of the right and left eye, measuring the horizontal distance from each eye to the picture centre, and recording the smaller of the two (Fig 1). All positions were normalized such that the horizontal centre was set to 0 and the horizontal endpoints of the image (whatever its aspect ratio) were equal to -0.5 and +0.5. The dependent variable was therefore the signed relative position of the most-centred eye, with a negative value signifying a position to the left of the physical centre. All measurements were taken using a program developed in R [13] to show images individually and record mouse click positions. The theoretical accuracy of these measures is therefore equal to one pixel on the screen used to display the image. Measurements were taken independently by two raters and averaged after checking for obvious inconsistencies, such as a right eye position to the left of the recorded position for the left eye. These resulted occasionally from rater errors in recording sequential clicks and were corrected by having raters repeat the measurement on the affected image. After correcting such errors, the overall inter-rater correlation was 0.971.

### Independent variables

Each image was tagged to identify the following variables: city of origin, taker’s sex, and selfie type. City of origin and sex were derived from the original database, whereas type was determined by inspecting each image individually. Each selfie was examined and classified as a “standard” or “mirror” selfie. A standard selfie was defined as a self-portrait taken by the sitter by holding a smartphone (or other suitable digital device) at arm’s length, such that the phone was not visible in the picture. A mirror selfie was defined as a self-portrait taken by photographing one’s own mirror image, such that the smartphone remained visible in the picture. As a consequence, mirror selfies are typically more likely to include larger parts of the sitter's upper body, with the head occupying less space in the picture and showing a different body pose. In addition, and more importantly for the purposes of the current analysis (as this may affect eye positioning), mirror selfies have been shown to be more likely to include a pose showing more of the right cheek (although this is in fact the left due to the mirror reflection), whereas standard selfies show the opposite trend [11]. Given these differences, it is important to include selfie type as a potential predictor of compositional choices.

## Results

**Fig 2:**
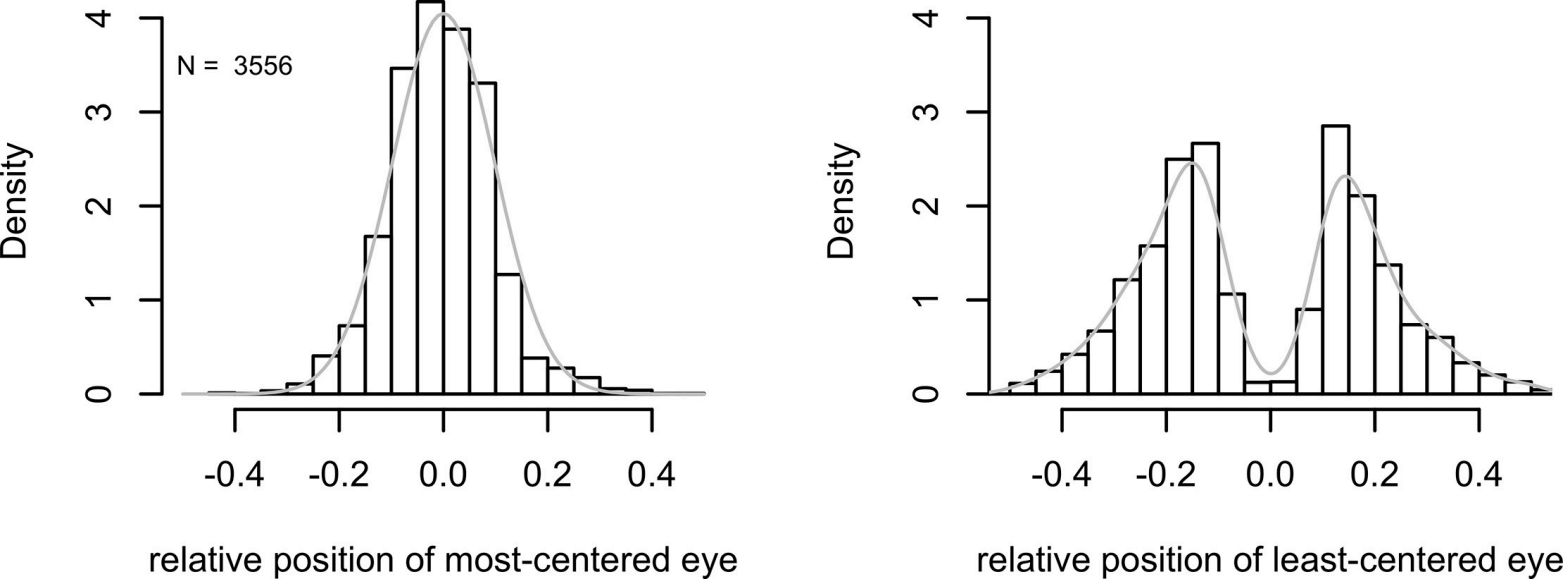
**Distributions of the positions of the most-centred (left) and least-centred (right) eyes in our selfie database.** Relative positions computed as as a proportion of frame width. Reference curves in grey are the Gaussian density function when μ = 0 and sigma = 0.1, which are the expected parameters given eye-centring and spread equal to the spread observed in our data (left), and density estimates based on the observed data (right).

The distribution of the position of the most-centred eye in our selfie database is presented in Fig 2 (left). Standard and mirror selfies are included in the same histogram as this distinction turned out not to predict differences in eye positioning (see following paragraphs). The distribution is leptokurtic (kurtosis = 4.2, Shapiro-Wilks test of normality W = 0.99, p < 0.001), with an excess of cases around the centre and relatively fewer cases in the tails. The standard deviation of the distribution is equal to 0.098. The central tendency of the distribution is very close to the horizontal centre of the picture frame for the selfies (position 0 on the histogram horizontal axis), although not exactly coincident with it. A 95% confidence interval around the arithmetic mean of this distribution yields a lower limit of -0.1 and an upper limit of -0.003, with an arithmetic mean of -0.007 of the picture width, which was therefore significantly different from 0, though less than 1%. This mean varied somewhat after subdividing the data according to the taker’s sex (males = -0.005, females = -0.007), to selfie type (standard selfies = -0.006, mirror selfies -0.011), and to city of origin (-0.002, -0.011, -0.006, -0.007, -0.009, -0.004 for Bangkok, Berlin, London, Moscow, New York City, and São Paulo, respectively). However, none of these differences proved statistically significant after subjecting the data to a 2 X 2 X 6 (sex by type by city) factorial Analysis of Variance (see ANOVA Table in S1 Table).

Importantly, the distribution of the least-centred eye was unambiguously bimodal (Fig 2, right), as one would expect if a tendency to centre one of the eyes affected pose choice. In addition, the distributions of the relative positions of the nose (mean = -0.013, SD = 0.135) and of the chin (mean = - 0.12, SD = 0.138) were significantly more variable than that of the most-centred eye (SD = 0.098), F(3564, 3564) = 7.1, p ⇒ 0.

We also tested whether there was a preference for centering one of the eyes. Indeed, participants chose to centre their left eye (N = 1931) more often than their right (N = 1625). When tested against the null hypothesis that the probability of choosing either eye is p = 0.5, these results also clearly yield a statistically significant difference, chi-squared(1) = 29.3, p ⇒ 0.

**Fig 3:**
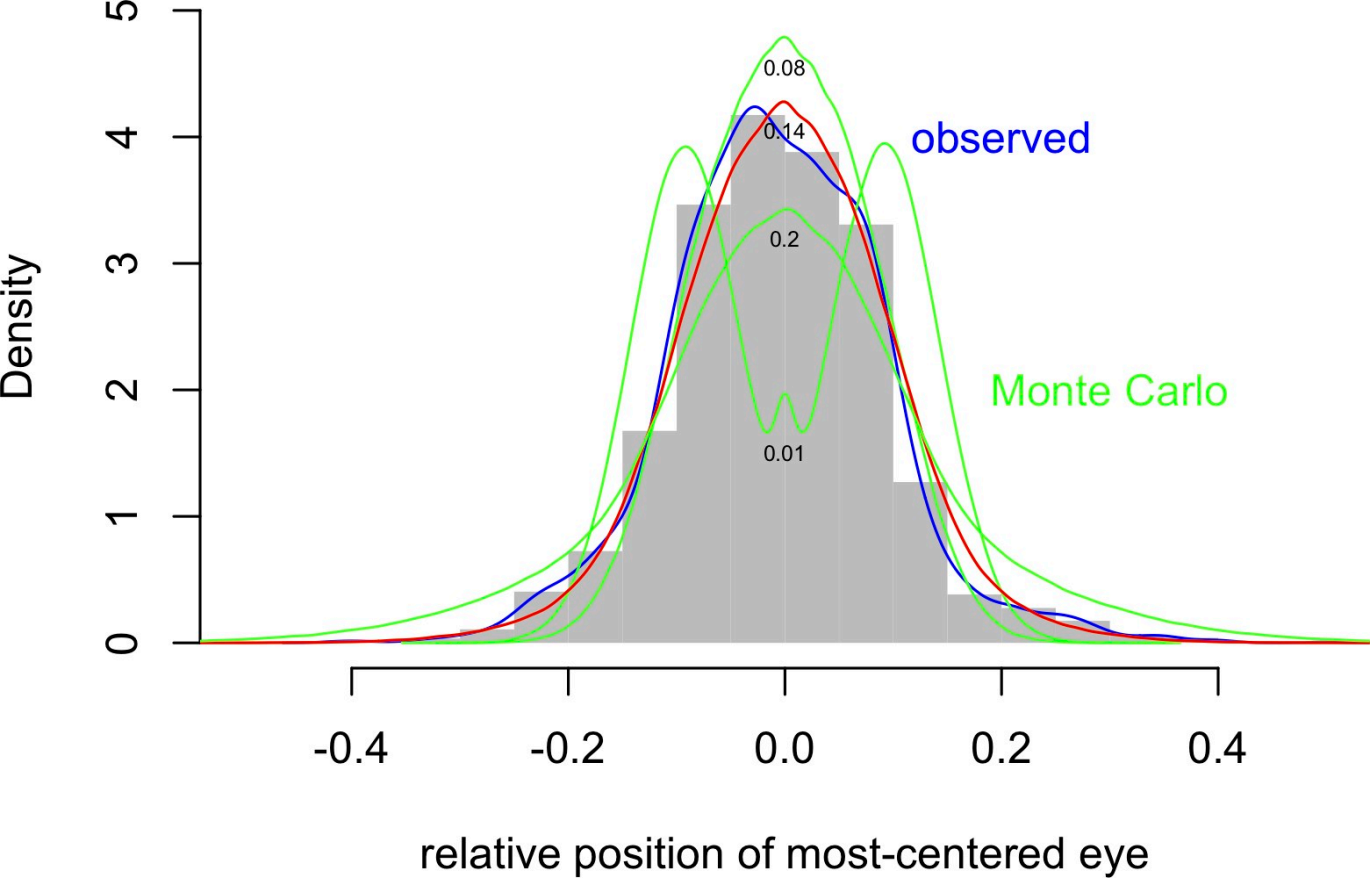
Densities of the simulated distributions of the most-centred-eye. Monte Carlo simulations in green and red (see text) plotted at four representative values of the standard deviation, σ (small numbers in black). The density (blue) of the observed distribution (histogram in grey) is plotted for comparison.

Finally, we compared the observed most-centred-eye positions to predictions based on Monte Carlo simulations of randomly placing the head within the picture frame. To compute simulated distributions, we drew simple random samples (n = 1 million) of head centre positions from theoretical normal distributions with μ = 0 and standard deviation = σ, where σ ranged from one-hundredth up to one-third of the picture width. Next, for each sample we drew another simple random sample from a second normal with μ = the observed average interocular distance in the selfies (20% of the total width of the picture) and σ = the observed standard deviation of the interocular distances in the selfies (10%). We then determined simulated positions for the left and right eyes by adding and subtracting half of the simulated interocular distances from the simulated face centres. This resulted in a set of simulated distributions of the most-centred eye, varying in shape as a function of σ. Specifically, very low values of σ yielded a bimodal distribution; higher values yielded unimodal distributions that changed from more leptokurtic to more platykurtic in comparison to the observed distribution as σ increased (see Fig 3). The best fit to the data (by a least-squares criterion) was obtained with σ at approximately 14% of the total width (red curve in Fig 3). Even in this case, however, the density of the simulated distribution showed non-negligible differences when compared to the observed density (blue curve in Fig 3), with an excess of data in the center relative to the tails and an overall shift to the left relative to zero. Accordingly, a test of goodness of fit (after binning the density vectors) resulted in a statistically significant difference, chi-square (8) = 30, p < 0.001.

## Summary, discussion, and conclusions

Selfies posted on *Instagram* provide evidence for eye-centring. Eye centring is observed in both males and female independently of the city of origin. Most interestingly, eye centring is observed both in standard and in mirror selfies. In addition, and importantly, eye centering is not perfect but shows a slight but statistically significant leftward bias of <1%. This bias is roughly consistent with the average values typically observed for pseudoneglect [8] and is remarkably consistent in all six cities of origin of the selfies. Finally, we observe that selfie takers tend to centre the left eye in the image more often than the right. We speculate that this preference may depend on the bias in favor of showing one's left cheek, which has been reported elsewhere for this very database [11–12] as well as other corpora of selfies [14–15] and in self-portraits by artists [16–17]. This conclusion however remains speculative due to an intrinsic limitation of the current database. Given that the images were freely posted by unknown users, we have no way of determining if some of them were left-right reversed before posting, or to estimate a proportion. We note however that a related bias was observed in our studies that used selfies taken on demand in our laboratory [14–15], where we had full control of the stored images. This is consistent with our speculation that the cheek bias reflects a preference for showing one's own left cheek rather that a preference for creating a image that *looks as if* the sitter were showing the left cheek. We emphasize however that this issue was not the main objective of the current study, which aimed at testing eye centring in the composition of the self-portrait.

The difference between our findings, which support eye centring, and previous results that did not [9] is likely to depend on the types of selfies that were analyzed. In our previous study, participants provided a selfie on demand as part of a laboratory study, and were instructed to hold the phone and search for a suitable pose according to predefined criteria. These constraints on the procedure for taking the selfie might have impacted the unconstrained composition of the image. Most important, in the earlier study participants could not alter or edit the selfie after taking it. When posting selfies on social media, instead, for many pictures it is likely that participants perform some kind of post-production processing before the selfie is uploaded. The most natural editing action, in particular, is likely to be cropping or otherwise altering the framing of the image, which would affect the position of the head and eyes within the picture frame. Another process that is likely to impact on the posted images is selection among several images. A selfie taker often will take several pictures and then compare them to choose one for posting. These forms of editing should be considered part of the visual composition of a selfie image, and therefore it could be argued that preventing them reduced the ecological validity of the images collected in the laboratory study, at least from the standpoint of composition.

A slight but consistent bias in centring to the left of the horizontal centre was evident for all cities, implying a residual pseudoneglect effect comparable to that seen in other tasks such as line bisection [8]. It may be objected that the negative average position of the most centred eye, which is consistent with a leftward bias, cannot be readily interpreted as a symptom of pseudoneglect because posing in most selfies is done while observing one’s self in the smartphone front-camera preview screen. Given that the preview screen reverses left and right (mimicking in part the optics of a mirror), if participants were trying to centre one eye on the pseudoneglect-dependent perceived centre, they would show a leftward bias in the preview image, but this would then correspond to a rightward bias in the saved picture as the saved picture in a smartphone is not mirror-reversed. This concern is related to the issue of left-right reversal mentioned above, and again suggests some caution in interpreting the finding.

We note however two things. First, this objection is valid for standard selfies, but not for mirror selfies as in this case the picture is taken with the back camera of the smartphone and faithfully replicates one’s own image in a real mirror. Interestingly, the leftward bias was, if anything, stronger in mirror selfies in comparison to standard ones. Second, this objection is valid for standard selfies only to the extent that composition is restricted to the evaluation of images in the preview screen. If saved selfies are then edited as suggested above, then any pseudoneglect effect should show up as a leftward bias in this *second* stage of composition. Thus, although it cannot be ruled out that at least for a fraction of standard selfies composition might have been based on the left-right reversed preview screen, it is more likely that in the majority of our analyzed selfies composition was based mostly on evaluating and editing the pictures however they were acquired, such that pseudoneglect would be expected to manifest itself as a leftward bias.

In conclusion, our results suggest that an eye-centring tendency may indeed be present when individuals compose a portrait. Evidence for this tendency is found not only in painted portraits and self-portraits by artists [2–3], but also in a large and ecologically valid sample of selfies, that is, of photographic self-portraits by individuals who are unlikely in the main to have received formal training in artistic composition. This in turn suggests that the eye-centring tendency depends on perceptual factors, possibly related to organizational principles governing our perception of balance in a composition, rather than on socio-culturally learned rules. Specifically, we observed that the most-centred eye had a narrower distribution relative to other candidate face features, such as the nose or the chin. We speculate that one eye is more naturally chosen as compositional center for horizontally positioning the face in the image as eyes are most informative about gaze direction and attentional state. These in turn may be used to infer the internal state, or mental content, of the individual depicted in the picture, thereby fulfilling a plausible communicative goal which may well apply to classic painted-self portraiture and contemporary selfies.

## Supporting information

S1 Table (DOCX)
