# Supplementary material for: Eye centring in selfies posted on Instagram
Source: PLoS One. 2019 Jul 17;14(7):e0218663. doi: 10.1371/journal.pone.0218663 (PMC6636715; doi:10.1371/journal.pone.0218663)
Supplement: S1 Table — (DOCX) [file pone.0218663.s001.docx]

Appendix

Summary of Analysis of Variance

dependent variable: relative position of most-centred eye

source DF SS MS F p

sex 1 0.00 0.004905 0.504 0.478

type 1 0.01 0.011808 1.213 0.271

city 5 0.03 0.006569 0.675 0.642

type 1 0.01 0.006619 0.680 0.410

sex x city 5 0.08 0.015090 1.551 0.171

type x city 5 0.02 0.003886 0.399 0.850

type x city 5 0.02 0.004237 0.435 0.824

Residuals 3532 34.37 0.009730
